# Forest-Scale Phenotyping: Productivity Characterisation Through Machine Learning

**DOI:** 10.3389/fpls.2020.00099

**Published:** 2020-03-06

**Authors:** Maxime Bombrun, Jonathan P. Dash, David Pont, Michael S. Watt, Grant D. Pearse, Heidi S. Dungey

**Affiliations:** ^1^Forest Informatics, Scion, Rotorua, New Zealand; ^2^Forest Genetics, Scion, Rotorua, New Zealand

**Keywords:** gradient boosting, decision trees, GPU-acceleration, LIDAR. forestry, phenotyping

## Abstract

Advances in remote sensing combined with the emergence of sophisticated methods for large-scale data analytics from the field of data science provide new methods to model complex interactions in biological systems. Using a data-driven philosophy, insights from experts are used to corroborate the results generated through analytical models instead of leading the model design. Following such an approach, this study outlines the development and implementation of a whole-of-forest phenotyping system that incorporates spatial estimates of productivity across a large plantation forest. In large-scale plantation forestry, improving the productivity and consistency of future forests is an important but challenging goal due to the multiple interactions between biotic and abiotic factors, the long breeding cycle, and the high variability of growing conditions. Forest phenotypic expression is highly affected by the interaction of environmental conditions and forest management but the understanding of this complex dynamics is incomplete. In this study, we collected an extensive set of 2.7 million observations composed of 62 variables describing climate, forest management, tree genetics, and fine-scale terrain information extracted from environmental surfaces, management records, and remotely sensed data. Using three machine learning methods, we compared models of forest productivity and evaluate the gain and Shapley values for interpreting the influence of categorical variables on the power of these methods to predict forest productivity at a landscape level. The most accurate model identified that the most important drivers of productivity were, in order of importance, genetics, environmental conditions, leaf area index, topology, and soil properties, thus describing the complex interactions of the forest. This approach demonstrates that new methods in remote sensing and data science enable powerful, landscape-level understanding of forest productivity. The phenotyping method developed here can be used to identify superior and inferior genotypes and estimate a productivity index for individual site. This approach can improve tree breeding and deployment of the right genetics to the right site in order to increase the overall productivity across planted forests.

## Introduction

Plantation forestry research seeks to optimise the productivity, profitability, health, and sustainability of commercial forests. This vital fibre supply system also provides many ecosystem services and is critical in meeting sustainable development goals to support the global population's increasing wood and fibre demands. The annual global fibre demand is expected to reach 11.4 billion m3 by 2050 and this cannot be extracted sustainably from the Earth's natural forests where growth rates are commonly as low as 2 m3/ha/y (Sedjo and Botkin, 1997). Intensively managed plantation forests must assume an increasingly prominent role in providing for the future demand in wood and fibre products. Increasing forest productivity whilst safeguarding forest health and sustainability will be critical to ensuring that this can be achieved (Sedjo and Botkin, 1997; Powers, 1999; Dash et al., 2019). Intensively managed forest systems such as the Southern hemisphere's *Pinus radiata D.Don* (radiata pine), and the South-Eastern USA's *Pinus taeda L*. (Loblolly pine) forests have been the subject of long-standing and detailed research programmes (Fox et al., 2007; Burdon et al., 2017). This research has helped to deliver improved productivity, profitability and helped to ensure wood fibre security.

Notable productivity increases have been achieved through genetic improvement (Kimberley et al., 2005), silvicultural intervention (Moore et al., 2018; Dash et al., 2019), and increasing site productivity through competition control (Richardson, 1993) and nutrient management (Will, 1980). The advent of the application of sophisticated remote sensing technologies to forest research has provided a new means for estimating numerous phenotypic traits through characterising the forest resource and providing a site description with unprecedented detail across large spatial extents (Pearse et al., 2017; Dash et al., 2017; Watt et al., 2019). This type of information can guide forest managers towards more comprehensive site specific management and provide an opportunity for precision deployment of improved genetic material. These datasets comprise a large number of observations and complex, highly-correlated variables meaning that traditional methods of analysis from forest research struggle to extract meaningful insights from them within practical time constraints to deliver meaningful findings. Combining these datasets with the emerging data driven approaches from the field of data science offers a new framework for extracting valuable insight that can help improve the productivity of plantation forests.

The application of high-throughput phenotyping to the agricultural sciences has accelerated realisation of gain from genetic improvement in many aspects of agronomy helping to secure the global food supply (Shakoor et al., 2017). In a similar manner, a framework for the application of high-throughput phenotyping to plantation forestry by incorporating remote sensing, genetic information, and site characteristics has been proposed (Dungey et al., 2018). These approaches require detailed quantitative phenotypic description of plant traits to be linked to information on the genetic composition of the system under study. Conventional phenotyping has been carried out manually and results in a bottleneck in data availability as it is costly, labour intensive, and technically challenging (Furbank and Tester, 2011; Araus and Cairns, 2014). Remote sensing offers a means by which phenotyping can be carried out across large areas and can provide detailed measurements of plant traits (Dungey et al., 2018). This approach could revolutionise the realisation of genetic gains and improve the understanding of the dynamics of key drivers of forest productivity. However, extending the phenotyping concept to the landscape scale is extremely challenging due to the large size of these datasets and the difficulty in disentangling the myriad of factors that influence growth across the forest landscape. As the specifics of the underlying model controlling the interaction between genetics and site factors is not completely understood, a data driven approach is appropriate.

When designing a model in domain-specific science, one strategy is to build a model from theoretical understanding and adjust its parameters based on the observed data until it fits with our interpretation of the process under study. Unfortunately, in many instances, such models are not well defined and the potential relationships between input variables are still under investigation and thus, unknown to the experts and researchers. The continual improvements of computational processing and algorithmic development have seen the advent of a new paradigm of data-driven modelling and the application of non-parametric machine learning techniques to build strong predictive models directly from the available data. One can consider building a large set of these models and combining them to obtain a stronger ensemble prediction. Neural network ensembles (NNs) (Hansen and Salamon, 1990) are one example of a machine learning method which can be combined in this manner. NNs are built from sophisticated algorithms that make them versatile, robust, scalable, and able to handle datasets with high dimensionality; however, these methods are generally slow and can be difficult to interpret. Support vector machines (SVM) (Drucker et al., 1997) are another class of machine learning algorithms that can be combined to handle complex nonlinear decision boundaries and guarantee a unique global solution for classification tasks; in recent years research interest in SVMs has waned among data scientists since the emergence of NNs. Random Forests (Breiman, 2001; Criminisi et al., 2012) rely on averaging of decision trees in the ensemble while gradient boosting methods (Natekin and Knoll, 2013) add new, weak models sequentially. Both are computationally efficient, provide clear insights into the impact of features (e.g. Gain and Shapley values) and the decision tree construction. They are able to deal with unbalanced and missing data yet they may over-fit on noisy data sets and cannot predict beyond the range of the training data.

Gradient Boosting Machine [GBM, (Friedman, 2001)] is a powerful tool in the field of supervised learning that achieves state-of-the-art performance on classification, regression, and ranking tasks. In a similar manner to Random Forests, the most popular implementations of gradient boosting combine the outputs from decision trees to build stronger predictors. Although decision trees are robust when handling numerical features, many data sets also include categorical features. These are discrete sets of values that are not necessarily comparable with each other (e.g. labels or nomenclatures) but may be equally as important for prediction as numerical features. Categorical features are commonly converted to numbers (ordinal encoding) before training the gradient boosting but some novel implementations have developed more efficient strategies including one-hot (Elman, 1990), binary, baseN, and mean encoding, or Bayesian encoders. While the rapid growth and ease of implementing GBM have given both academics and practitioners new ways of engaging and solving problems (Lawrence et al., 2004; Foucard et al., 2011; Torlay et al., 2017), this speed of adoption has not been followed by the development of clear guidelines to select algorithms and implementations to use according to data set properties (prediction, classification, sparsity, dimensionality).

Building on a previously developed conceptual framework (Dungey et al., 2018) for a forest phenotyping platform, in this paper we seek to develop an advanced analysis pipeline for integrating phenotypic traits with genetic and site information across a major plantation forest. We compared three state-of-the-art implementations of gradient boosted decision trees (GBDTs) XGBoost (Chen and Guestrin, 2016), LightGBM (Ke et al., 2017), and CatBoost (Dorogush et al., 2018) to model forest productivity as a function of both numerical and categorical features. Specifically, we measured the model training and prediction times, as well as the root-mean-square error score (RMSE) and the coefficient of determination (*R*^2^) for the testing and the training data sets. Thus, we were able to identify the fastest model with the best accuracy that was most robust to noise.

## Methodology

### Study Site and Features of Interest

**Figure 1:**
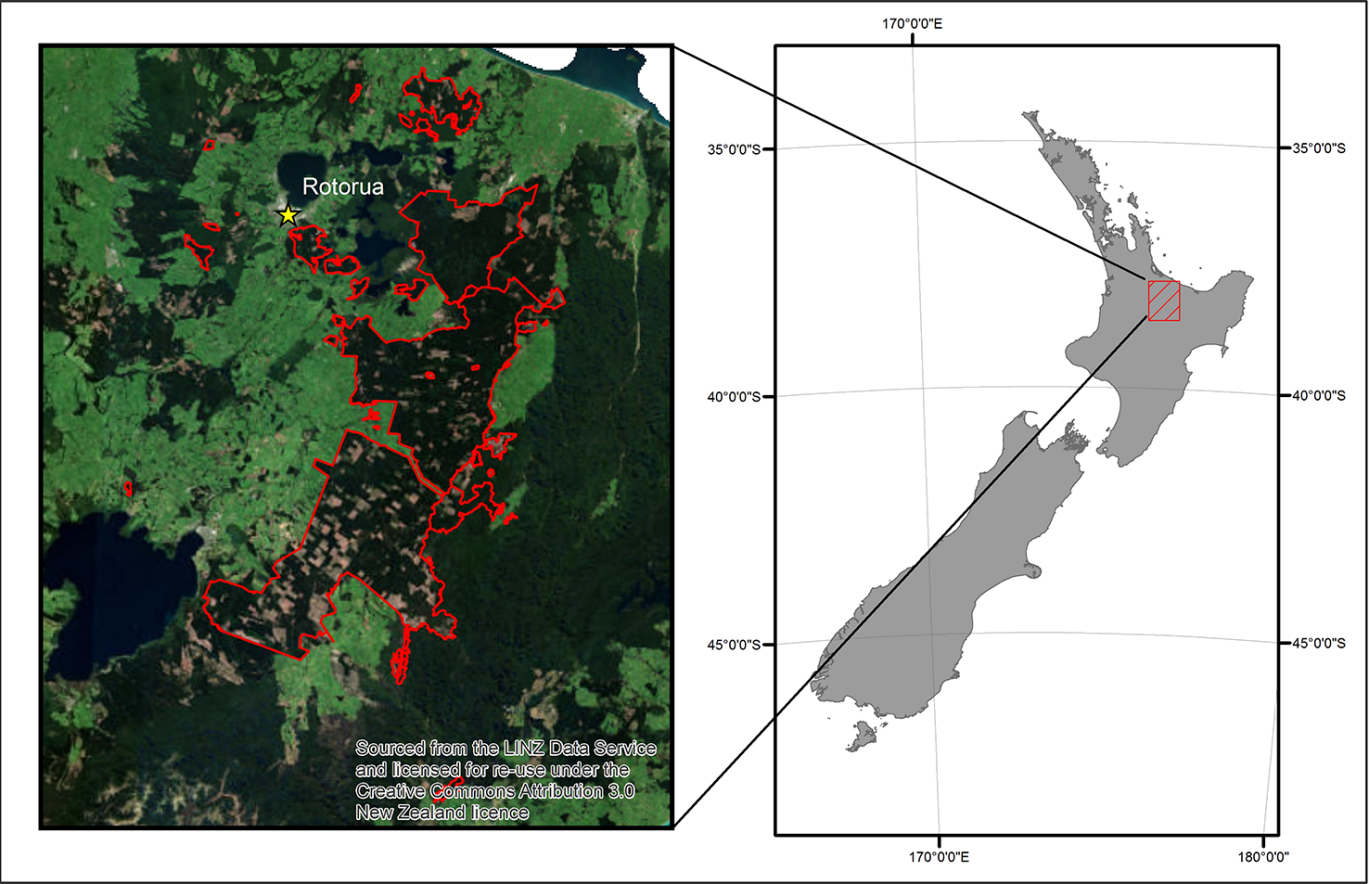
The study forest location within New Zealand and the extent of the ALS data outlined in red.

The data were collected from Kaingaroa forest which is located in the central North Island region of New Zealand (**Figure 1**). The study was restricted to stands of *P. radiata*, which cover 90% of the ∼180 000 ha of the forest where all the features were interpolated to a 25 m resolution. This resolution is equivalent to the size of the measured field plots used in the models of productivity (see eq. 1), and could be improved by developing models at tree-level but this would require a totally different approach.

The methods used to derive the analytical data set were detailed in (Dungey et al., 2018) and are briefly summarised here for the convenience of the reader. The forest managers provided a geo-spatial database describing the silvicultural operations (**Table A3**) and soil records. This database included the tree species, initial and current stand density, pruning and thinning status, soil classification (Hewitt et al., 2010) and carbon to nitrogen ratio (C/N). The Radiata Pine Breeding Company (RPBC, Rotorua, New Zealand) provided information on seedlot identities and associated growth and form (GF) ratings, and an estimate of the genetic performance of each seedlot within the forest (**Table A1**).

A large variety of climatic variables (e.g., annual and seasonal averages for temperatures, rainfall, wind speed, sunshine hours, and total global radiation see **Table A2**) were extracted from national surfaces generated by the New Zealand crown research institute National Institute of Water and Atmospheric Research (NIWA) and clipped to the extent of the Kaingaroa forest. Finally, an airborne laser scanning (ALS) survey and field measurement dataset were processed to extract topographical features (detailed in **Table A3**) (Bahner and Antoni, 2009) and derived features such as visible sky, valley depth (Rodriguez et al., 2002), wind exposure (Gerlitz et al., 2015) and wetness index, and tree phenotypic traits such as tree height, Site Index (Watt et al., 2015), and leaf area index (LAI) (Pearse et al., 2017).

### Field and Remote Sensing Data

Systematic sampling without replacement was employed to locate field plots throughout the study forest. In total 500 plots were located at the intersections of a grid that had a randomised start point and orientation and were measured between the 1st March and 8th August 2014. The sampling unit was a slope adjusted 0.06 ha bounded, circular field plot. A survey grade global navigation satellite system (GNSS) was used to fix the centre of each plot. Within each field plot diameter at breast height (dbh) was measured for all trees. Tree height was measured for a subset of plot trees, selected from across the dbh range, that were free from excessive lean or malformation. These field measurements were used to calculate Site Index.

An ALS survey was undertaken between the 23rd January and 6th March 2014 using an Optech ALTM 3100EA Pegasus scanner to collect a discrete, small-footprint dataset. Data collection was characterised by a pulse rate frequency of 100 kHz, a maximum scan angle of 12° off-nadir, and a swath overlap of 25%. These settings yielded a data set with a footprint size of 0.25 m and a mean pulse density of 11.5 points m2. Returns were classified into ground and non-ground returns automatically using the TerraScan module of the TerraSolid software product (Terrasoid, Espoo, Finland). Classification accuracy was improved by subsequent manual inspection and reclassification where required. Metrics extracted from the ALS data included height percentiles (P5ht, P10ht, P20ht,…, P99ht, m), the mean (Hmean, m) and maximum height (Hmax, m), several metrics describing return height distribution through the canopy [skewness, coefficient of variation, standard deviation (SD) and kurtosis] and measures of canopy density, and pulse penetration, such as the percentage of returns reaching within 0.5 m of the ground (Pzero, %) and the percentage returns above 0.5 m (Pcover, %). These descriptive variables providing information on the canopy structure were extracted from the ALS data and were used in combination with the field plot data to model the phenotypic trait Site Index across the study forest.

#### Derivation of Site Index

The response variable used in this study was Site Index. Site Index for *P. radiata* in New Zealand is defined as the mean top height at age 20 years (Goulding, 2005). Field data was used to fit a regression between dbh and measured tree height and this was then used to predict the heights of unmeasured trees within each plot. This information was used to calculate mean top height (MTH), defined as the average height of the primary leaders of the 100 largest diameter trees per hectare. This measure of MTH was used to estimate Site Index (SI) by rearranging the following equation:

(1) $$MTH=0.25+(SI-0.25)\left[\frac{exp(-aT)}{1-exp(20a)}\right]\frac{1}{b0+b1SI}$$

where T = age (years) taken from stand records and a = a0 + a1L + a2E, where L = Latitude (°S) and E = Elevation (m). Model coefficients were taken from a national height age model for *P. radiata* in New Zealand (van der Colff and Kimberley, 2013).

### Mapping Phenotypic Variation

An estimate of phenotypic variation across the landscape was mapped through developing a spatial surface of forest productivity. The parametric modelling methods described in (Watt et al., 2015; Watt et al., 2016) were used to describe the distribution of Site Index across the forest based on the ALS data set and Site Index extracted from the field plots described above. The purpose of this process was to provide a response variable that can potentially be linked to genetic and environmental factors across the study forest.

### Gradient Boosting Methods

Gradient Boosting Machine (GBM) is a supervised learning algorithm. Using a set of labelled training data as an input, it builds a model that aims to correctly predict the value of each training instance based on other information referred to as the features of the instance. GBM creates a strong model by sequentially combining weak models generated from a gradient descent algorithm over an objective function. This objective function optimisation is held out in the function space where the function increments are the “boosts” and the weak learners are the “base-learners”. The base-learners can include Markov random fields (Dietterich et al., 2004), wavelets (Viola et al., 2001), linear models, and decision trees. Decision Tree (DT) learning is a method that develops a model by repeatedly splitting subsets of the training instances. These methods produce interpretable models that are useful for a wide range of problems. Maximum performance is achieved when many trees are combined into an ensemble model. The ensemble then returns for each estimate, the value that appears the most often (i.e., the mode) of all predictions, thus providing better accuracy by reducing the variance of the estimate.

These favourable properties support the use of DT as a base learner for the three GBDTs compared in this study where we examined the eXtreme Gradient Boosting (XGBoost), the CatBoost method (for categorical boosting), and the LightGBM. XGBoost (Chen and Guestrin, 2016) was released in March 2014 as a successor to the Multiple Additive Regression Trees method (Friedman and Meulman, 2003). This method maintained the interpretability of the tree boosting approach whilst offering faster computation times (Simple/limited/incomplete benchmark for scalability, speed and accuracy of machine learning libraries for classification, 2017) and more robust regularisation based on the Newton descent (Wright and Nocedal, 1999; Rebentrost et al., 2016). Subsequently, LightGBM (Ke et al., 2017) was developed in January 2017 and brought novel techniques for splitting, more efficiently than the histogram-based algorithm of XGBoost and for handling categorical variables. The approach was later revisited and enhanced with unbiased gradients calculated not on the current model, like in classical GBDTs, but through random permutations as implemented in CatBoost (Dorogush et al., 2018) in April 2017.

The three implementations grow and prune their trees differently and certain hyperparameters vary between the GBDTs trialled. For example, XGBoost's *min_child_weight* (i.e., the minimum sample size at one node to decide either to stop or keep splitting) is not defined in the CatBoost or LightGBM algorithms, while some hyperparameters have different limitations. CatBoost's *depth* parameter is restricted to between 1 and 16 but is without restrictions for the other methods. To provide a fair comparison we carefully selected hyperparameters that have similar functionality (regulation, iteration, depth/wide) for all GBDTs tested (**Table 1**).

**Table 1:** Time and score comparisons for the training of the three GB methods on the training set.

	XGBoost	CatBoost	LightGBM
	max_depth: 4	depth: 5	max_depth: 4
	learning_rate: 0.05	learning_rate: 0.1	learning_rate: 0.1
Hyperparameters	min_child_weight: 3	l2_leaf_reg: 5	num_leaves: 10
	n_estimators: 6,877	iterations: 10,369	n_estimators: 6,571
		one_hot_max_size: 10	
Training Time	119 sec	518 sec	406 sec
RMSE	1.6968	1.6385	1.6736
*R*^2^	0.86	0.87	0.87

#### Categorical Features

A total of 62 features were used in this analysis of which eight were categorical variables. These included features describing the silvicultural management of the trees (e.g. thinning and site preparation methods), type of seedling storage (containerised, bareroot) and breeding methods (open/control pollination) at the nursery, the type of genetic improvement (clonal/non-clonal and seedlot identifier) and the NZ soil classification identifier. Unlike CatBoost and LightGBM, XGBoost can only accommodate numerical values and categorical features must be encoded manually during data preparation. LightGBM uses a special algorithm, faster than one-hot encoding (Elman, 1990) to partition the value of categorical features specified by their indexes. Under this approach, the histogram of a categorical feature is sorted according to its accumulated values (*sum_gradients/sum_hessian*) and then the best split on the sorted histogram is found according to the training objective at each split (Fisher, 1958). CatBoost uses two methods to encode categorical features. The categorical features with a number of different labels less than or equal to the given one_hot_max_size parameter are encoded using one-hot encoding. The remaining categorical features are transformed by quantisation by computing statistics (usually average or median of the response) on random permutations of the dataset and clustering the labels into new classes with lower cardinality [see Eq. 1 in (Dorogush et al., 2018)].

#### Model Implementation and Evaluation

We developed scripts to implement the three GBMs using Python 3.7.3 (van Rossum, 1995) on the Ubuntu 16.04 operating system with 12 Intel^®^ Core^TM^ i7-8700K CPU @ 3.70GHz and 32GB Memory. We used the GPU-accelerated versions of the three GBMs (Mitchell and Frank, 2017; Zhang et al., 2017; Dorogush et al., 2018), supported by an NVIDIA GeForce GTX 2080 GPU. In our workflow, the overall steps for implementing a regression tree model are as follow:

1. Processing missing and categorical values;
2. Split into training and testing sets;
3. Use the training set to tune the hyperparameters;
4. Train a model on the training set and evaluate the error on the testing set;
5. Train a model on all data for interpretation and estimation/prediction.

The first step of the data pre-processing was the conversion of the Not-a-Number (NaN) values to a large negative integer (e.g., -1,000) to be i) understandable by the three methodologies and ii) separated from the lowest observations (close to zero). A copy of the dataset is created from which the observations without the dependent variable are removed, reducing the number of observations from 2746851 to 2311918 (i.e., 84% still present). To avoid any misconversion from string/float to integer, and since XGBoost handles only numerical values while CatBoost and LightGBM encode categorical values as part of their implementations, the categorical features were coded as an integer. Then, to minimise overfitting while retaining randomness and fair representation (and because we have a large dataset), we used a shuffled, stratified split (train_test_split function from sklearn library (Pedregosa et al., 2011) to partition the dataset into a training set (70%) and testing set (30%) that are used for hyperparameter tuning and evaluating prediction error from the trained model on the testing set, respectively.

As hyperparameter tuning using a conventional grid search is an extremely computationally intensive process, we developed an efficient oriented hyperparameter tuning process. Hyperparameter tuning was achieved through the implementation of an oriented grid search in which the optimised parameter set is selected iteratively. During the first tuning iteration, the first hyperparameter is evaluated over its entire range of values using 3-fold cross-validation (3-CV) based on the RMSE score. The best hyperparameter values are then retained for the next tuning iteration. During subsequent tuning iterations, the original combinations are then evaluated over the current hyperparameter range to select a new best hyperparameter set. Therefore, for *p* hyperparameters of each range *n_p_*, instead of having $\prod_{i=1}^{p}n_{p}$ tuning iterations for grid search, this stage is reduced to *n*_1_(*n_p_*-1) number of iterations which significantly reduces the computational load of the hyperparameter tuning process.

Due to the complexity of the data set and the noise resulting from extrapolating and merging spatial data from various modularities (remote sensing, climate stations, etc.), the *early_stopping* argument which halts training when the score does not improve, does not provide a robust solution against overfitting. This is because the RMSE score continues to slowly decrease for a great number of trees (i.e., iterations) and/or depth. This results in a tendency to overfit the model without significant improvement in model predictive accuracy. To overcome this whilst maintaining some stochasticity in the future estimations and predictions, we integrated a condition on the best iteration being the step where

(2) test_rmse_mean−train_rmse_mean>0.1

is verified, so that a variability of 10% is authorised between the average RMSE score of the 3-CV between the training (*train_rmse_mean*) and the testing (*test_rmse_mean*) sets.

A model was then fitted to the training data with the best hyperparameters set from the lowest iteration that satisfied the condition of Eq. 2. Using this model, a prediction for the testing set was developed to validate the model based on its accuracy and robustness. To assess model accuracy, we calculated the coefficient of determination (*R*^2^, see Eq. 3), i.e., the proportion of variance in the dependent variable that is predictable from the features, and the RMSE (Eq. 4), this being the average deviation of the fitted values (*f_i_*, i.e., predicted values) from the observed values (*y_i_*).

(3) $$R^{2}=1-\frac{\sum_{i}{\left(y_{1}-f_{i}\right)}^{2}}{\sum_{i}{\left(y_{i}-\bar{y}\right)}^{2}}$$

Where *ȳ* is the mean of the observed values.

(4) $$RMSE=\sqrt{\frac{1}{N}\sum_{i=1}^{N}{\left(y_{i}-f_{i}\right)}^{2}}$$

Where *N* is the number of observations.

Finally, we use the entire data set to fit an original model with the best hyperparameters set from the lowest iteration that met the condition in Eq. 2. This final model was used to estimate the missing observations removed during the preprocessing step and to evaluate the direct impact of some features on the forest productivity.

We investigated the influence and interactions of the key features at both a global and a local level. At a global level, we extracted the most important features with the greatest predictive power and characterised the importance of these features with the “Gain” metric [also called Gini Importance or Mean Decrease in Impurity (Breiman, 2017)]. This metric reveals the relative contribution of the corresponding feature to the model by summing the improvement in accuracy (or gain) per split for each decision tree in the model. At a local level, we can identify which features are most important for each individual prediction in the context of the other feature values. For example, while the impact of temperatures might be highly influential for the entire forest, trees growing at higher altitudes might be most strongly influenced by the elevation or aspect of the growing site. To explore these local influences, we used the Shapley values [equation 5 and (Shapley, 1953; Lundberg and Lee, 2017)] that calculate the importance of a feature by comparing model prediction with and without the inclusion of the feature of interest. Shapley values were calculated as

(5) $$\phi_{i}=\sum_{S\subseteq F/i}\frac{|S|!(|\text{F}|-|S|-1)!}{|F|!}(f_{S\cup i}-f_{S})$$

where *ϕ_i_* is the Shapley value of a feature *i* (from the set of features *F*). At a high level, interpretation of equation 5 calculates the difference between model prediction with [(*f* (*S* ∪*i*)] and without [(*f* (*S*)] the feature of interest *i*. Effectively, the method retrains the model on all feature subsets *S* ⊆ *F*, the change in prediction quantifies the impact of the feature. This is done in every possible order to keep the comparison of features fair since the order in which a model is exposed to features can affect its importance. Therefore, the final Shapley additive explanation (SHAP) values arise from averaging the *ϕ_i_* values across all the possible orderings.

The code for the proposed productivity models and GBM comparison is available at https://github.com/maxBombrun/forestPhenotyping.

## Results

### Model Development and Hyperparameter Tuning

Hyperparameters for all three GB methods investigated were successfully tuned and the final model was fitted to the experimental dataset. The hyperparameters selected through the tuning process were similar for all three GB models. The optimal max_depth ranged between 4 and 5 while the learning rate selected varied between 0.05 for XGBoost to 0.1 for both CatBoost and LightGBM (**Table 1**). Only XGBoost has a min_child_weight parameter and this was tuned to a value of 3. All three models included a hyperparameter for the number of estimators and although we tried to keep them in the same range, the values verifying Eq. 2 varied between models with 6,877 for XGBoost, 10,369 for CatBoost, and 6,571 for LightGBM (**Table 1**).

Overall, the training RMSE and *R*^2^ scores were very similar for the three models tested (**Table 1**). XGBoost was faster for training and prediction, but the model was slightly less accurate, most likely due to the fact that the categorical variables were not encoded. The training of CatBoost took almost two minutes longer than LightGBM and four times longer than XGBoost. This was because of the high number of iterations needed to converge with the optimal set of hyperparameters. However, CatBoost exhibited the best model performance, producing the lowest RMSE and highest *R*^2^ values for the training data. LightGBM produced the highest *R*^2^ score for both training and validation and had an acceptable time for training and predicting, yet the longest for the latter.

### Model Validation

Model validation showed that all three models provided highly-accurate predictions of Site Index (**Table 2**). The LightGBM model produced the highest *R*^2^ value (0.88) followed by CatBoost (0.86) and XGBoost (0.85). CatBoost produced the lowest value of RMSE (1.71 m) followed by LightGBM (1.73 m) and XGBoost (1.76 m). Prediction times for all three models were very fast and the best performance was achieved using XGBoost (0.76 sec) followed by CatBoost (14 sec) and LightGBM (19 sec).

**Table 2:** Time and score comparisons for the validation of the three GB methods on the testing set.

	XGBoost	CatBoost	LightGBM
Prediction Time	0.76 sec	8 sec	19 sec
RMSE	1.7595	1.7137	1.7344
*R*^2^	0.85	0.86	0.88

### Model Interpretation

**Figure 2:**
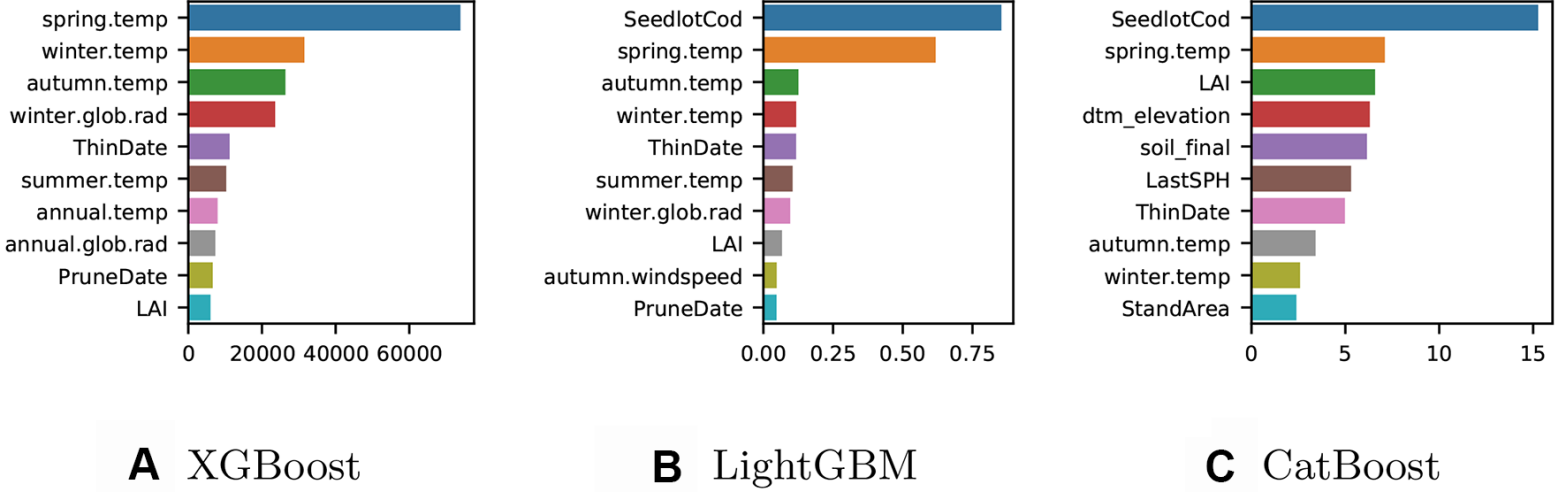
**(A)** XGBoost gain: number of observations affected by the splits based on a feature. **(B)** LightGBM gain: total sum of gain on prediction from the splits based on a feature **(C)** CatBoost gain: average gain on prediction from the splits based on a feature.

We trained a final model with all the available data for each implementation and computed the gain to provide insight into the relative importance of each feature. Although each method implemented showed similar “split-based” measures of gain, the gain scores were not directly comparable due to slight differences in the way these are calculated. Gain for XGBoost is influenced by the count of the number of samples affected by the splits based on a feature (**Figure 2A**), for LightGBM the total gain of splits which use the feature is summed (**Figure 2B**), while for CatBoost gain values show for each feature, how much on average the prediction changes if the feature value is permuted (**Figure 2C**).

The ten most important features varied somewhat between the three different models. The XGBoost model (**Figure 2A**) does not include any of the categorical variables in the top ten features of importance, while information on the genetic identity (*seedlotCod*) appears in the most important features for both CatBoost and LightGBM and the soil classification (*soil_final*) appears in the five most important features of CatBoost. For the XGBoost model, the most important predictors were features related to the climatic conditions such as seasonal temperatures and global solar radiation. Features describing LAI and silvicultural intervention were also included in the ten most important predictors for XGBoost. The climatic conditions were also highly important in the LightGBM model (**Figure 2B**) although the Seedlot ID (*seedlotCod*) was the most important feature for predicting Site Index. The most accurate model was CatBoost (**Figure 2C**) and the most important predictors for this model were seedlot ID (*seedlotCod*), spring temperature and LAI followed by a series of predictors relating to the terrain, soil classification (*soil_final*), silvicultural intervention, and other seasonal temperatures.

**Figure 3:**
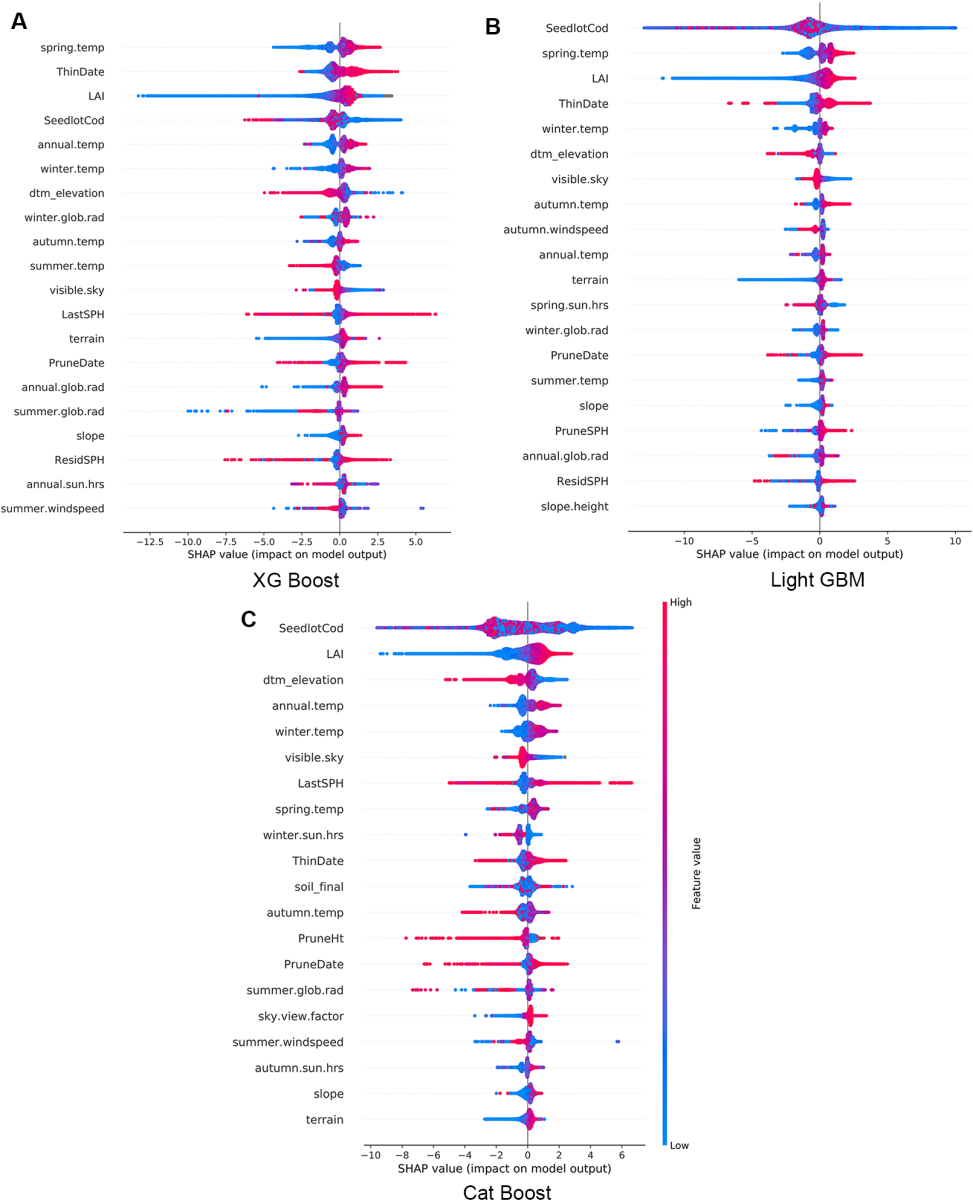
Impact of variables for XG Boost **(A)**, Light GBM **(B)**, and Cat Boost **(C)**. Every observation has one dot in each row. The position of the dot on the x-axis is the impact of that feature on the model's prediction for the observation, and the colour of the dot represents the value of that feature for the observation.

Using the final models, we calculated and plotted the Shapley values (SHAP) for every observation across the study forest. In **Figure 3** the features are sorted by the mean magnitude of the associated SHAP value. In these figures, each datum represents one observation, its colour is related to the actual value of the feature (blue for low values and red for high values) while the position on the x axis shows the impact, i.e., difference between prediction and observation, which is positive (respectively negative) when the feature generates improvement (respectively deterioration) in the prediction.

The three models consistently show that high LAI has a small positive impact on Site Index, but low values predominantly have a large negative impact on Site Index (**Figure 3**). Some observations are correlated to a small positive impact demonstrating the interaction with other features within the model. Similarly, terrain elevation (*dtm_elevation*) is inversely proportional to productivity - the lower the elevation the more positive was the impact on Site Index. In contrast, spring air temperature had a proportional correlation where low temperatures have a negative impact and higher temperatures have a positive impact on Site Index.

### Productivity Estimation and Prediction

**Figure 4:**
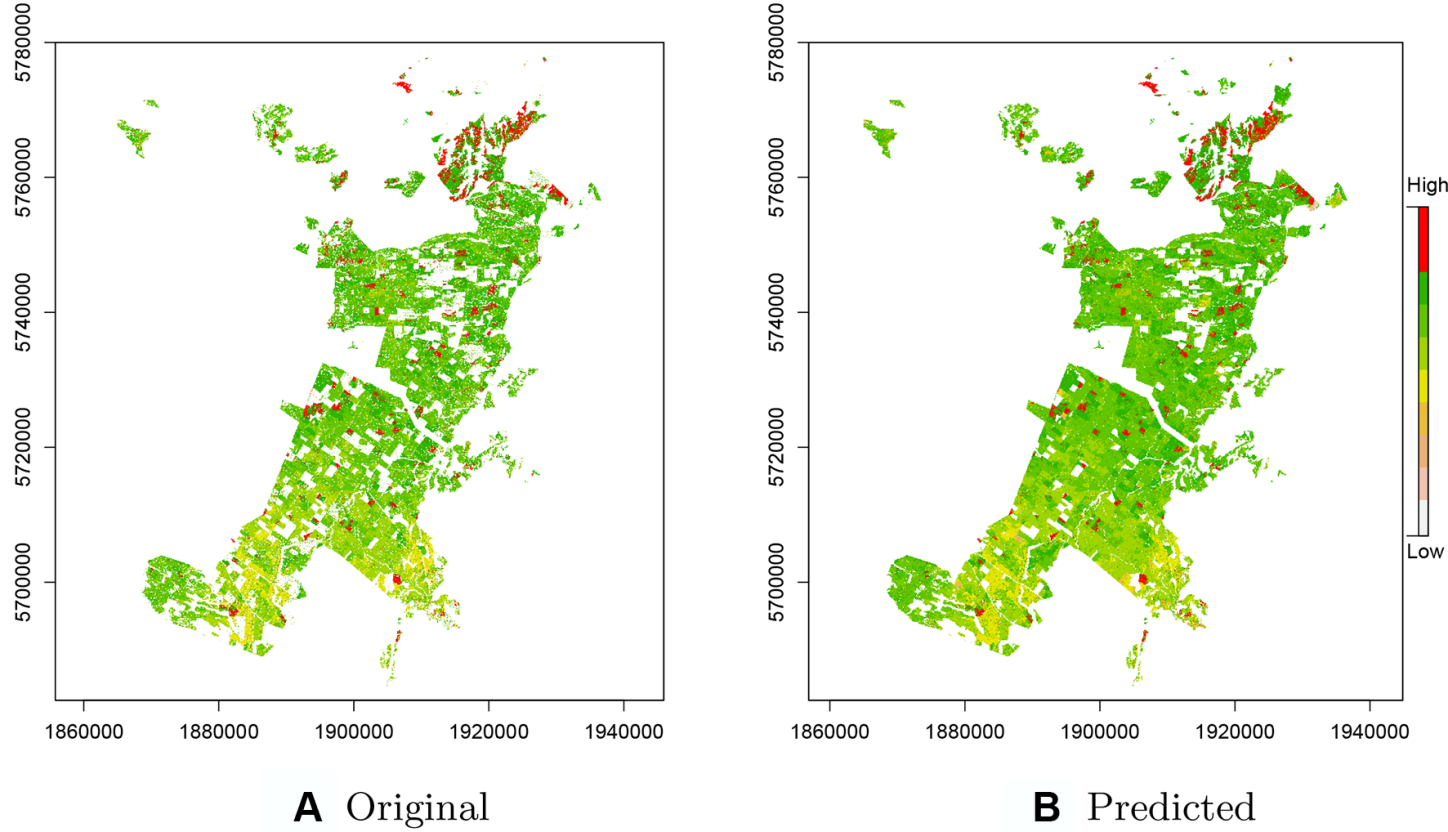
**(A)** Original raster layer of the Site Index across the study forest with missing values. **(B)** Predicted raster layer of the Site Index based on the CatBoost model.

As CatBoost provided the most precise predictions we used this implementation for prediction of productivity across the entire original data set including the ∼435k missing observations removed during the pre-processing step (**Figure 4**). These predictions included areas for which there were no initial estimates of Site Index as these were unproductive or unplanted areas, or areas planted in other species. Comparisons of these estimates with the original Site Index surface, derived from ALS data, show a high level of correspondence (**Figure 4B**). The predictions accounted for both the overall increase in productivity from south to north and also were able to detect the fine scale high productivity hotspots throughout the forest (see red areas—**Figure 4B**).

**Figure 5:**
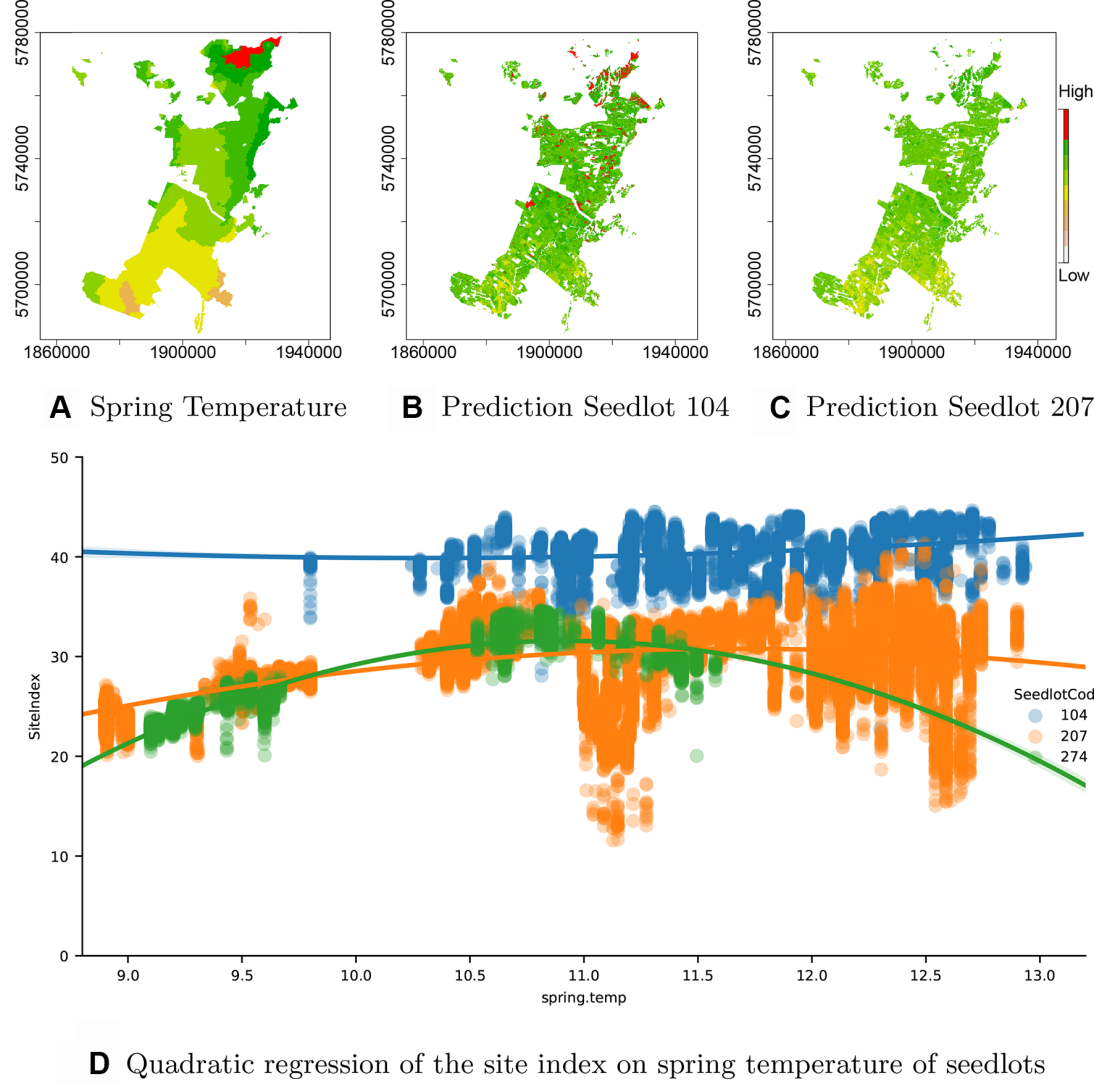
Interaction of GxE. **(A)** Spring temperature across the forest showing an increase from south to north (orange: low to red: high), **(B)** spatial variation in Site Index for Seedlot 104 and **(C)** Seedlot 207. Also shown is **(D)** the relationship between spring air temperature and Site Index for Seedlots 104 (blue), 207 (orange) and 274 (green).

As the *seedlotCod* feature is robustly encoded by CatBoost, an estimation of productivity can be obtained from the model for any *seedlotCod* that is well represented across the estate. This is demonstrated through predictions of Site Index for the highest productivity seedlot, Seedlot 104 and two seedlots with the lowest productivity, Seedlots 207 and 274 (**Figure 5D**)^1^.

Seedlot 104 had a markedly higher mean Site Index (40.6 m) than either Seedlot 207 (29.3 m) or 274 (26.2 m). Interestingly, the high Site Index of Seedlot 104 was very consistent across the range of spring air temperatures over which it occurred and a fitted polynomial showed very little curvature over this range (**Figure 5D**). In contrast, Site Index for clone 274 demonstrated a marked quadratic relationship with air temperature increasing from ca. 22 m at 9.2°C to an optima of ca. 32 m at a spring air temperature of ca. 11.0°C (**Figure 5D**). Similarly, Site Index for Seedlot 207 increased to an optima around 11.5°C but did not markedly decline at spring air temperatures above this optima (**Figure 5D**).

Spatial predictions of Site Index for Seedlot 104 (**Figure 5B**) demonstrate the relative stability of Site Index for this seedlot across the temperature gradient (**Figure 5A**) found throughout the forest. In contrast model predictions for Seedlot 207 demonstrate a broader range in Site Index which more closely reflects the south to north gradient in spring air temperature.

## Discussion

Our principal aim was to implement a robust model capable of handling a large, forest-scale (2.7 million observations) dataset with complex (undetermined interactions) and noisy (disparate acquisitions) features with mixed data types (categorical/continuous) to predict forest productivity. Furthermore, we sought to develop robust procedures to tune and select the optimal model for our data set and to understand the interaction between the key drivers of productivity. To successfully achieve this objective, we investigated three recent implementations of the GBM machine learning algorithms, XGBoost (Chen and Guestrin, 2016), LightGBM (Ke et al., 2017), and CatBoost (Dorogush et al., 2018). Across the estate, Site Index was derived for 2.3 million observations (84% of total forest) using data from an ALS survey and field plot measurements. These were split into a training set to fit the models and a testing set to validate and compare the model performance in terms of both computation time and model accuracy (**Tables 1** and **2**). In this manner, we were able to successfully model Site Index using the experimental data set with a robust and interpretable modelling approach. As the assembled data set was large and complex it was critical that we carefully considered the computing time when suggesting a modelling approach. The modern GBMs assessed were able to fit predictive models in a timeframe that was practically feasible from an operational perspective. These promising results indicate that the forest phenotyping approach we have presented and explored shows significant potential for improving forest productivity, accelerating the realisation of gains from tree breeding programmes, and furthering the domain of forest research.

A secondary objective of our study was to examine the performance of the various GB models tested both in terms of accuracy and computational performance. The model fitted by the XGBoost method was the fastest for both the training (∼4 times) and prediction (∼8 times) times; however, this method exhibited the highest RMSE and the lowest R^2^ scores making it the least accurate of the three models tested. This is likely due to the basic ordinal encoding of the categorical features, which is faster to complete but not representative of decision thresholds for these features which are therefore less used for splitting (**Figure 2A**).

Thanks to the histogram-split method (Fisher, 1958) for encoding the categorical features, the LightGBM offers the best R^2^ scores for training and validation of the three methods, but just slightly better than XGBoost regarding the RMSE. It also requires longer times to train the model and exhibited the slowest time for prediction which is only 19 seconds.

The newest of the three implementation, the CatBoost method (Dorogush et al., 2018), offered the best compromises. Although its training time is slightly longer than the other algorithms, it has the lowest error score for both training and prediction. The flexibility to choose multiple encoding based on the one_hot_max_size parameter (set up to ten in our model, see **Table 1**) allowed us to use one-hot encoding for the categorical features with low cardinality (here below ten), and use quantisation to encode the categorical features with higher cardinality (here 22 for *soil_final* and 1106 for *seedlotCod*). This benefit is evident in **Figure 2C** where both *seedlotCod* and *soil_final* are amongst the top ten features of importance for predicting Site Index.

The data set used in this study is composed of 62 variables (61 features, plus Site Index) derived from high precision technology (e.g., ALS survey), human inputs (silvicultural and inventory field measurements) and permanent monitoring (climate stations). Our processing pipeline purposely does not integrate a feature reduction step although previous studies have shown that this step can improve model performance and improve computation times (Criminisi et al., 2012). We followed this approach firstly because, unlike DT methods, boosting approaches do not randomly select correlated features in each tree (which, in DT, creates a 50%/50% importance for two highly-correlated features), thus ensuring GBMs handle multicollinearity correctly (Tianqi et al., 2019). Secondly, by keeping all the features, our data-driven study can robustly inform us about the importance of all features explored, according to three different models. We inferred feature discrimination at a global level (38 features have a gain lower than 1 and amongst these 16 have a gain lower than 0.5 such as nursery stock type and thinning type) and feature dependencies at a per-observation level (**Figure 5D**) representative of the GxExS interactions. Our approach could be reproduced on new forests with less data by excluding some of the less important features. The features which provided the lowest gains might be time consuming, or dangerous to collect or estimate and might introduce bias through their inclusion. Nonetheless, it is important to note that some of the less important features had the highest number of missing values, making it important to confirm the significance of the features with domain experts.

The investigation of the features of importance (**Figure 2**) demonstrates the high value of the encoding for categorical features. *seedlotCod* is important in the three models but a prediction based on partial order decision could adversely affect the output. As a result we confidently recommend the encoding approach discussed and followed in this study. The influence of genetics was strong in both the LightGBM and CatBoost models and, along with key environmental variables, was a significant factor impacting productivity. We observed significant variation between seedlots across the environmental range within the forest. This was used to map variation in productivity between seedlots at a fine spatial scale under varying environments for the study forests using the final model. This novel approach provides new insight into the impact of the interaction between genotype and site factors on productivity within the plantation forests.

We found that Site Index was also highly sensitive to seasonal air temperature in the study forest. This finding is consistent with previous research using both process-based and empirical forest productivity models that showed air temperature to be the most important regulator of New Zealand grown *P. radiata* height (Kirschbaum and Watt, 2011), Site Index (Hunter and Gibson, 1984; Watt et al., 2010) and volume (Watt et al., 2010). Previous studies show an optimal temperature for growth which is reached at a mean annual temperatures of between 12–15°C (Watt et al., 2010). This optimum range is broadly comparable to the results in our study which showed optimal Site Index to be reached at spring air temperatures of ca. 11°C. The broad agreement of the findings presented here with previous research, and our understanding of the factors affecting forest productivity, indicates that the approach developed is producing outputs that accurately represent the biological system under investigation.

Our modelling approach may provide valuable information for optimising the deployment of seedlots for current conditions and as climatic conditions change. Using this method the continual optimisation of deployed genetically improved tree stock across the forest can be used to respond to emerging risks (e.g. novel pathogens or increased drought) and opportunities provided by changing growing conditions. For example, the increased air temperature expected under climate change could favour the further deployment of clone 104 as this clone appears to have high productivity at warmer air temperatures (**Figure 5**). Removal of the poor performing seedlots from future deployment will help to lift overall forest productivity ensuring that the wood and fibre supply from the forest can be secured and improved. In a similar manner, genotypes or seedlots that consistently perform well can also be identified, and increased deployment of these to targeted sites will help to improve forest productivity.

## Conclusion

In this study, we have developed and optimised a processing pipeline for a data-driven forest phenotyping platform using a state-of-the-art machine learning approach. Remote sensing methods such as ALS can now provide numerous candidate phenotypic variables, at high-resolution, across forest sites. Such data sets comprise large numbers of observations, and variables, many of which are often highly correlated. It is rapidly becoming intractable to apply traditional modelling methods to such data. Data science methods, such as the model described here, can provide a viable approach to analyse this data and derive useful system insights.

Following investigation of three gradient boosting machines, we found that CatBoost offered the greatest model precision and acceptable computation performance for our requirements. Through harnessing most of the available information within the forest this model allowed us to quantify the impact of genetics on forest productivity and how genetics interacts with environment. The outputs from this model provide great insight into how environment regulates productivity and give the forest manager the means of increasing productivity through more closely matching seedlots with their preferred sites. Further research should acquire a broader range of qualitative data (e.g., branching, straightness, wood density) for different genotypes in order to characterise more comprehensively the genetic traits dynamically affected by the interactions between genetic, environmental, and silvicultural factors.

## Data Availability Statement

The datasets generated for this study will not be made publicly available. The datasets are based on commercially sensitive information and also include indigenous data.

## Author Contributions

MB has developed and implemented the machine learning approaches and wrote the core text of the manuscript. JD has pre-processed the data and worked on the previous version of the algorithm. DP, MW and GP have worked on the remote sensing methodology and extract relevant data for the study. HD has developed the genetic approach and expertise required for the study. All the authors have participated in the writing and the review of the manuscript as well as providing relevant expertise in forestry and their specific field of science.

## Funding

This research was undertaken as part of the Growing Confidence in Forestry's Future research programme, which is jointly funded by the New Zealand Ministry of Business, Innovation and Employment (contract No C04X1306) and the Forest Growers Levy Trust.

## Conflict of Interest

The authors were employed by the New Zealand Forest Research Institute Limited, trading as Scion.

The authors declare that the research was conducted in the absence of any commercial or financial relationships that could be construed as a potential conflict of interest.

## Footnotes

1 To protect proprietary information all codes were re-assigned a random integer.

## Appendix A Features of the dataset

**Table A1:** Genetic variables of the dataset.

GxExS trend	Feature name	Description
Genetic	SeedlotCod	Categorical variable representative of the seed family.
Genetic	Clone	Categorical variable of two classes encoding the condition “the tree is a clone”.
Genetic	GF	Growth and form score.

**Table A2:** Environment variables of the dataset.

GxExS trend	Feature name	Description
Environment	SiteIndex	In New Zealand, mean top height at age 20 years derived from Eq. 1.
Environment	Temp^2^	Mean temperature in degree Celsius per day.
Environment	glob.rad	Amount of accumulated global solar radiation in MJ/m^2^.
Environment	sun.hrs^2^	Number of hours of sun per day.
Environment	tot.rain^2^	Total amount of rain in mm per day.
Environment	windspeed^2^	Mean wind speed in m/s at 10m above ground level over 24 hours.
Environment	Aspect^3^	Local morphometric terrain parameters derived from multi-scale fitting based on (Bahner and Antoni, 2009).
Environment	dtm_elev^3^	Elevation of the terrain in metres calculated from the digital terrain model.
Environment	mid.slope.position^3^	Mid-slope position is assigned with 0 whereas maximum vertical distances to the mid-slope in both valley or crest directions are assigned with 1.
Environment	normalised.height^3^	Normalised height allots value 1 to the highest and value 0 to the lowest position within a respective reference area (Bahner and Antoni, 2009).
Environment	sky.view.factor^3^	Calculation of visible sky based on (Bahner and Antoni, 2009).
Environment	slope.height^3^	Difference in altitude between the pixel and the local channel.
Environment	slope^3^	Local morphometric terrain parameters derived from multi-scale fitting based on (Bahner and Antoni, 2009).
Environment	standardised.height^3^	Product of normalised height multiplied with absolute height.
Environment	Terrain	Automated classification of topography calculated from the DTM. Based on the algorithm presented in (Iwahashi and Pike, 2007).
Environment	valley.depth	Valley depth calculated using the Top Hat algorithm presented in (Rodriguez et al., 2002).
Environment	vector.ruggedness	Vector ruggedness calculated following the algorithm presented in (Sappington et al., 2007).
Environment	visible.sky	Estimate of visible sky calculated using the Top Hat algorithm presented in (Rodriguez et al., 2002).
Environment	wetness.index	Topographic wetness index calculated using (Moore et al., 1991).
Environment	wind.exp	Topographic assessment of wind exposure calculated based on the algorithm presented in (Gerlitz et al., 2015).
Environment	cn_rk5	Carbon to nitrogen ratio representative of the fertility.
Environment	LAI	Leaf area index, a dimensionless quantity that is defined as the one-sided leaf area per unit ground area. Derived using the methods defined in (Pearse et al., 2017).
Environment	soil_final	Categorical variable representative of the soil composure following the New Zealand Soil Classification (Hewitt et al., 2018).

^2^These features are split into 5 variables representing annual, summer, autumn, winter, and spring averages over 30 years.

^3^Feature extracted from SAGA GIS (http://www.saga-gis.org/en/index.html) based on the DTM.

**Table A3:** Silviculture variables of the dataset.

GxExS trend	Feature name	Description
Silviculture	StandArea	Area of the stand.
Silviculture	Crop.Init.SPH	Stand density at which seedlings were established.
Silviculture	Rotation	The number of successive replantings that occurred on this stand.
Silviculture	ThinClass	Categorical variable representative of the thinning management regime such as number of thinning or crown release.
Silviculture	ThinType	Categorical variable representative of the management method for thinning such as production thinning, waste thinning and waste thinning with low pruning.
Silviculture	ResidSPH	Residual from the inventory of the stand per hectare after harvesting.
Silviculture	PruneClass	Categorical variable representative of the pruning (removal of lower branches) management regime.
Silviculture	PruneSPH	The stand density of pruned stems.
Silviculture	PruneHt	Height (in m) to which the tree has been pruned.
Silviculture	MaxDOS	Refers to the maximum diameter of the stem at the point of pruning, including branch stubs.
Silviculture	LastSPH	The stand density taken during the last inventory of the stand prior to harvesting.
Silviculture	ThinDate	Date of the last thinning.
Silviculture	PruneDate	Date of the last pruning.
Silviculture	Seedlot.Planting.Stock	Categorical variable representative of the type of management in the nursery such as control and open pollination, clonal cuttings and plantlets or seedling top cutting.
Silviculture	Seedlot.Planting.Stock.Type	Categorical variable representative of the type of stock, this being container, bareroot or plug seedlings.
